# Cellobionate production from sodium hydroxide pretreated wheat straw by engineered *Neurospora crassa* HL10

**DOI:** 10.1007/s00449-024-03061-w

**Published:** 2024-07-12

**Authors:** Jiajie Wang, Takao Kasuga, Zhiliang Fan

**Affiliations:** 1 Department of Biological and Agricultural Engineering, University of California , Davis, One Shields Avenue, Davis, CA 95616 USA; 2 Department of Plant Pathology, University of California , Davis, One Shields Avenue, Davis, CA 95616 USA; 3 United States Department of Agriculture—Agricultural Research Service, University of California, Davis, CA 95616 USA

**Keywords:** Cellobionic acid, Wheat straw, *Neurospora crassa*, Sodium hydroxide pretreatment

## Abstract

This study investigated cellobionate production from a lignocellulosic substrate using *Neurospora crassa* HL10. Utilizing NaOH-pretreated wheat straw as the substrate obviated the need for an exogenous redox mediator addition, as lignin contained in the pretreated wheat served as a natural mediator. The low laccase production by *N. crassa* HL10 on pretreated wheat straw caused slow cellobionate production, and exogenous laccase addition accelerated the process. Cycloheximide induced substantial laccase production in *N. crassa* HL10, enabling the strain to yield approximately 57 mM cellobionate from pretreated wheat straw (equivalent to 20 g/L cellulose), shortening the conversion time from 8 to 6 days. About 92% of the cellulose contained in the pretreated wheat straw is converted to cellobionate. In contrast to existing methods requiring pure cellobiose or cellulase enzymes, this process efficiently converts a low-cost feedstock into cellobionate at a high yield without enzyme or redox mediator supplementation.

## Introduction

Due to the rising global energy crisis and pressing environmental issues, there is a growing interest in producing fuels and chemicals from renewable resources to replace those derived from petroleum [1, 2]. Organic acids directly made from sugars have drawn substantial attention, for organic acids and their derivatives are essential platform chemicals [1, 2]. An aldonic acid is produced via the oxidation of the aldehyde functional group in an aldose to the corresponding carboxylic acid. Among them is lactobionic acid (LBA), which is comprised of a galactose moiety linked to a gluconic acid molecule. LBA is obtained from the oxidation of lactose. LBA displays biodegradable, antioxidant, biocompatible, anti-aging, and chelating properties and has wide applications in the food, pharmaceutical, chemical, and cosmetic industries [3–7]; thus, it is well-positioned as a high-value, low-volume chemical [7]. Cellobionic acid (CBA) is a stereoisomer of lactobionic acid composed of a glucose moiety linked to a gluconic acid molecule instead. Because of the physicochemical similarity of CBA and LBA, CBA is expected to have similar applications as those of LBA [8, 9]. Therefore, cellobionate could potentially serve as an alternative carbon source for fuels and chemical production. A study conducted by Desai et al. demonstrated the production of iso-butanol from the cellobionate [10]. Tao et al. investigated using cellobionate and glycerol as the co-substrate to produce ethanol [11].

CBA (cellobionate) can be produced by the oxidation of cellobiose. Oh, et al. produced cellobionate from cellobiose using *Pseudomonas taetrolensas,* which homologously expressed a quinoprotein glucose dehydrogenase. The cellobionate production titer and productivity were 200 g/L and 9.52 g/L/h, respectively [12]. Using the same recombinant strain, they further improved productivity by adopting a strategy of whole-cell catalysis [13]. However, in both cases, refined cellobiose was needed as the substrate. Oh, et al. utilized the same strain as a whole-cell catalyst to produce LBA using lactose as the substrate [14]. Because cellobiose is a much more expensive starting material than lactose, it leads to a higher production cost of cellobionate than the cost of producing lactobionate. To lower the feedstock cost, Yoo et al. investigated the possibility of producing CBA using waste paper as the substrate [15]. Under optimized reaction conditions, cellulase-catalyzed hydrolysis generated 23 g/L cellobiose from 80 g/L waste paper. CBA was produced at a final titer of 24 g/L from the 23 g/L cellobiose using the recombinant *P. taetrolens* strain [15]. However, the addition of cellulase enzymes for the hydrolysis of waste paper to produce cellobiose introduces an additional processing cost.

In previous studies, we engineered *Neurospora crassa* strains to produce cellobionate from cellulose without adding any enzyme [8, 16]. The wild-type *N. crassa* strain can naturally produce cellulases that hydrolyze cellulose to form cellobiose [17]. The engineered *N. crassa* strain F5*∆ace-1∆cre-1∆ndvB* has six β-glucosidase*(bgl*) genes and the cellobionate phosphorylase (*ndvB*) gene deleted from the genome of the wild-type strain [16]. These deletions disabled the strain’s ability to consume cellobionate or cellobiose produced from cellulose hydrolysis. In addition, the transcription factors genes *acre-1* and *cre-1* were also deleted, which led to higher cellulase production on cellulose. This strain produced 20 mM cellobiose and 10 mM cellobionate from 20 g/L Avicel in 7 days [16].

Cellobiose dehydrogenase (CDH) is the key enzyme to convert cellobiose to CBA or lactose to LBA [18]. When CDH oxidizes cellobiose to form CBA, CDH accepts the electrons and gets reduced. The electrons carried by the reduced CDH need to be transferred to an electron acceptor for CDH to regain functionality [19]. The re-oxidation of CDH by oxygen is slow and is the rate-limiting step of CBA and LBA production. The CDH-redox mediator-laccase system has been widely used to improve the conversion of lactose to LBA [19–23]. In such a system, artificial redox mediators such as dichlorophenolindophenol (DCPIP) or 2,2’-azino-bis 3-ethylbenzothiazoline-6-sulphonic acid (ABTS) can reoxidize the reduced CDH, then get regenerated through oxidation by laccase. Laccase then passes the electrons to oxygen as the final electron acceptor. The CDH-redox mediator-laccase system was also used to improve cellobionate production from cellulose. When laccase and a low concentration of ABTS were added to the fermentation system, the strain *N. crassa* F5*∆ace-1∆cre-1∆ndvB* produced about 40 mM cellobionate from Avicel [24]. To avoid exogenous laccase addition, Lin et al. heterologously expressed a codon-optimized *Botrytis aclada* laccase gene in the *N. crassa* F5∆*ace-1*∆*cre-1*∆*ndvB* strain to enable laccase production under a copper-inducible producer, resulting in the strain *N. crassa* HL10. This strain could convert cellulose to cellobionate without the addition of any enzyme. About 81% of the cellulose was converted, and the titer produced was 47.4 mM. The yield of cellobionate from the consumed cellulose was approximately 94.5% [8]. However, a catalytic amount of ABTS was still required to achieve efficient conversion while pure cellulose (Avicel) was used as the substrate.

In the present study, we are interested in converting wheat straw, a sustainable and low-cost cellulosic feedstock, to cellobionate using the engineered *N. crassa* strain HL10. Wheat straw is the main by-product of wheat production. It is the second most abundant agricultural residue in the U.S. Wheat straw, like other cellulosic biomass, contains cellulose, hemicellulose, and lignin as the three major components. It needs an effective pretreatment process to facilitate the hydrolysis of the cellulose contained by fungal cellulases. Leading pretreatment methods include acid or base pretreatment, steam explosion, and ammonia explosion treatment [25, 26]. Among these, sodium hydroxide (NaOH) pretreatment is one of the most common pretreatment methods that has been extensively studied in processing cellulosic biomass [27]. Compared to dilute acid-based pretreated wheat straw, NaOH-pretreated wheat straw effectively dissolves lignin and retains more hemicellulose fraction in the pretreated solids [27]. We report the conversion of NaOH-pretreated wheat straw to cellobionate using *N. crassa* HL10, identification of the conversion bottlenecks, and strategies to improve the conversion.

## Material and methods

### Strain and chemicals

The strain *N. crassa* HL10 used in this study was constructed previously in the lab [8]. Wheat straw was obtained from the Idaho National Laboratory, which contained 34% cellulose, 18% hemicellulose, and 16% lignin (Idaho Falls, ID, USA). ABTS, DCPIP, cellobiose, sodium fluoride, β-glucosidase from *Aspergillus niger*, laccase from *Aspergillus sp.*, cycloheximide, p-nitrophenyl-β-D-lactopyranoside (pNPL), were obtained from Sigma Aldrich.

### Diluted sodium hydroxide pretreatment

Wheat straw was first milled and sieved to pass through meshes of size 0.45 – 1 mm. The milled wheat straw was pretreated in 2% NaOH solution at 121 °C for 30 min with 10% w/v solid loadings. After pretreatment, the pretreated solids were washed with a large quantity of water. The pH of the washed solids was adjusted to 7 using concentrated H_2_SO_4_ before being dried in a 70 °C oven for 48 h. The contents of glucan, xylan, and lignin were determined according to the standard protocol described in NREL/TP-510–42618 [28].

### *N. crassa* HL10 submerged fermentation experiments

*N. crassa* HL10 was grown on agar with 1 × Vogel’s media and 2% sucrose for 3 days in an incubator with light at 30 °C and another 7 days at room temperature to produce spores. Fermentation was carried out in 250 mL Erlenmeyer flasks in a rotary shaker at 200 rpm with constant light at 30 °C for 10 days. Each flask contained 1 × Vogel’s media, 3 g/L glucose, 20 g/L Avicel or pretreated wheat straw with 20 g/L cellulose equivalent, and 0.8 mM CuSO4. The total liquid volume is 50 mL. Fermentation was initiated by inoculating the flasks with 10–14 days old conidia at a final ${OD}_{420}$ of 0.1. Samples were collected at various time intervals to measure cellobiose and cellobionate concentrations. To study the role of ABTS and laccase in cellobionate production, a final concentration of 0.02 mM ABTS or/and 0.5 U/mL laccase was added to the fermentation broth at the specified time. To study the effect of cycloheximide addition time on cellobionate yield, cycloheximide was added at a concentration of 3 μM on day 2. All fermentation conditions were performed at a minimum in triplicate.

### Enzyme activity measurement

CDH and laccase assay were conducted using previously established methods, with slight modifications [16, 29]. CDH activity was determined by measuring the reduction of DCPIP in 96 well plates using a plate reader at 515 nm at 30 °C (${\upvarepsilon }_{515}=\text{6,800 }{\text{M}}^{-1}{\text{cm}}^{-1}$). Cellobiose was used as the substrate. The final assay mixture contained 0.25 mM DCPIP, 75 mM sodium acetate buffer, 3.75 mM cellobiose, and 5 mM sodium fluoride. One unit of CDH activity is defined as the amount of enzyme required to reduce 1 μmol of DCPIP per minute.

Laccase activity was measured by the oxidation of ABTS (${\upvarepsilon }_{436}=\text{29,300 }{\text{M}}^{-1}{\text{cm}}^{-1}$) at 436 nm at 30 °C in a plate reader. The assay mixture contained 2 mM ABTS and 150 mM sodium acetate buffer. 10 mg/ml β-glucosidase from *Aspergillus niger* was added to the sample at a volumetric ratio of 1:1 to hydrolyze all the cellobiose contained in the sample to glucose, removing the substrate for CDH to oxidize before the laccase assay to avoid interference from CDH activity in the laccase assay. One unit of laccase activity is defined as the amount of enzyme required to oxidize 1 μmol of ABTS per minute.

Exoglucanase (CBH) activity was carried out using pNPL as the substrate following a previous protocol with slight modifications [8]. The reaction mixtures containing 80 μL of 1 mg/mL pNPL in 50 mM citric acid buffer (pH 4.8) and 80 μL of five folds diluted culture supernatants were incubated at 37 °C for 30 min. The reaction was then quenched by adding 80 μL of 150 mM NaOH to the solutions. The released p-nitrophenyl was measured at 405 nm. One unit of CBH activity is defined as the amount of enzyme required to release 1 μmol of p-nitrophenyl per minute. Nitrophenyl is used to prepare for the standard curve.

### Fungal biomass measurement

Fungal growth estimation was achieved by evaluating glucosamine content with a modified version of a previously described method [30]. Fermentation residue, along with filter paper, underwent sequential washing with 15 mL of acetone and 30 mL of DI water. The washed samples were then mixed with 9 mL of 1.2 g/mL potassium hydroxide. Hydrolysis was carried out by autoclaving at 121 °C for 40 min. To precipitate glucosamine, 25 μL of the resulting hydrolysate was combined with 0.5 mL of ice-cold 70% ethanol, followed by the addition of 0.15 mL of Celite suspension (1 g of Celite suspended in 10 ml of 70% ethanol). After keeping in ice for 20 min, the samples were centrifuged at 12,500 rpm for 30 min at 0 °C and the supernatant was removed. This procedure was repeated by an initial wash with 0.8 mL of ice-cold 40% ethanol and two subsequent washes with 0.8 mL of ice-cold DI water. Finally, glucosamine content was determined through a colorimetric assay in accordance with previous studies. Standard curves were prepared with glucosamine hydrochloride (0–30 μg/mL) [30].

### Cellobiose and cellobionate analysis

Concentrations of cellobiose and cellobionate were measured by a Shimadzu high-performance liquid chromatography (HPLC) at 80 °C equipped with a CARBOSep COREGEL 87C column (Concise Separations, San Jose, USA) and refraction index detector (RID). 4 mM calcium chloride was used as the mobile phase at a flow rate of 0.5 mL/min [16].

### Calculations of the yields

The cellulose conversion was calculated by the amount of cellulose consumed divided by the starting cellulose amount. The xylan conversion was calculated by the amount of xylan consumed divided by the starting xylan amount. The cellobionate yield from consumed cellulose was defined as cellobionate produced (molar) divided by the amount of cellulose consumed (molar, assuming the molecular weight of 324 g/mol).

### Statistical analysis

All experiments were performed in triplicates. The analyzed data are presented as the mean ± standard deviation (SD) of three triplicates. Statistical analysis was performed using Microsoft Excel.

## Results and Discussion

### Composition of the alkali-pretreated wheat straw

 The NaOH-pretreated wheat straw has the following composition: glucan 55.9 ± 2.9%, xylan 20.3 ± 1.0%; and lignin 13.2 ± 3.0%.

### The exogenous addition of ABTS on cellobionate production on Avicel by *N. crassa* HL10

**Fig. 1:**
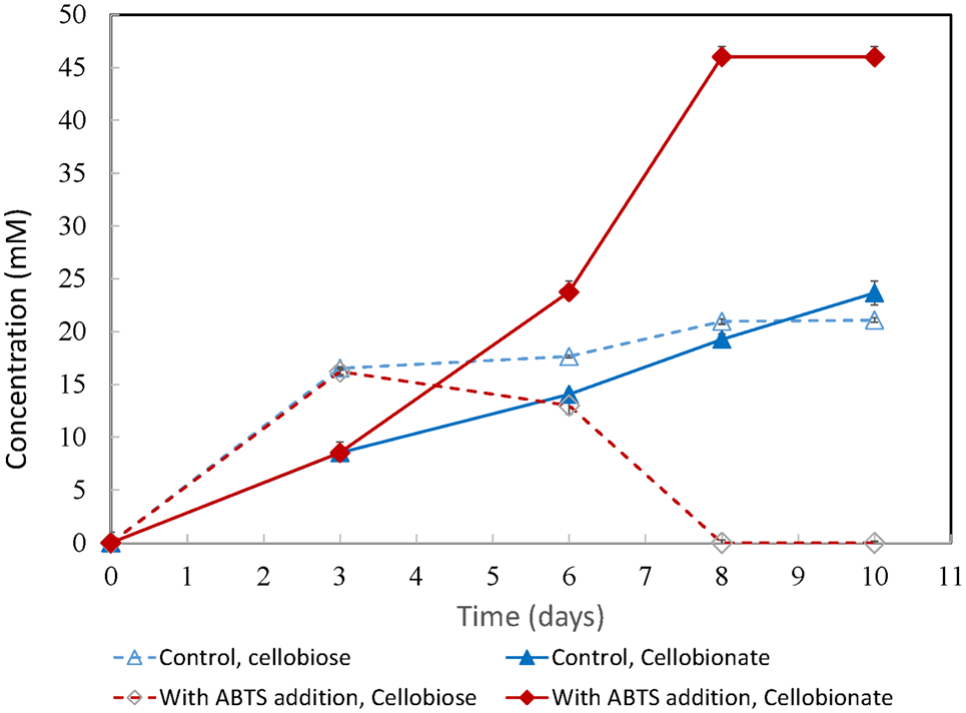
The effect of ABTS on cellobionate production from Avicel using *N. crassa* HL10. The experiments were conducted in triplicate. Error bars indicate the standard deviations of sample replicates

The effect of redox mediator (ABTS) on cellobionate production using Avicel (20 g/L) as the substrate was investigated. ABTS was added to fermentation flasks on day 3 to reach a final concentration of 0.02 mM. Cellobiose and cellobionate concentrations were tracked for ten days. As shown in Fig. 1, cellobiose accumulated to 16 mM on day 3, indicating that the rate of cellobiose production from cellulose hydrolysis was faster than the rate of conversion to cellobionate. Without ABTS addition, the cellobiose and cellobionate concentrations kept rising throughout the experiment. The titers of cellobionate and cellobiose reached 23 mM and 21 mM at the end of 10 days, respectively. The presence of ABTS caused a deacrease in the cellobiose concentration after day 3 and an increase in cellobionate concentration at a higher rate than without ABTS addition. On day 8, the cellobionate concentration was about 47 mM, and cellobiose was completely consumed. Adding a catalytic amount of ABTS doubled the cellobionate titer. Since *N. crassa* HL10 produces both CDH and laccase [8], the CDH-redox mediator-laccase system in the HL10 fermentation seemed to be redox mediator limited when grown on Avicel. Supplementing exogenous redox mediator substantially improved the cellobionate titer and productivity. Although ABTS was only needed in catalytic amounts, it is costly for industrial applications.

### The effect of ABTS addition on cellobionate production by HL10 on pretreated wheat straw

**Fig. 2:**
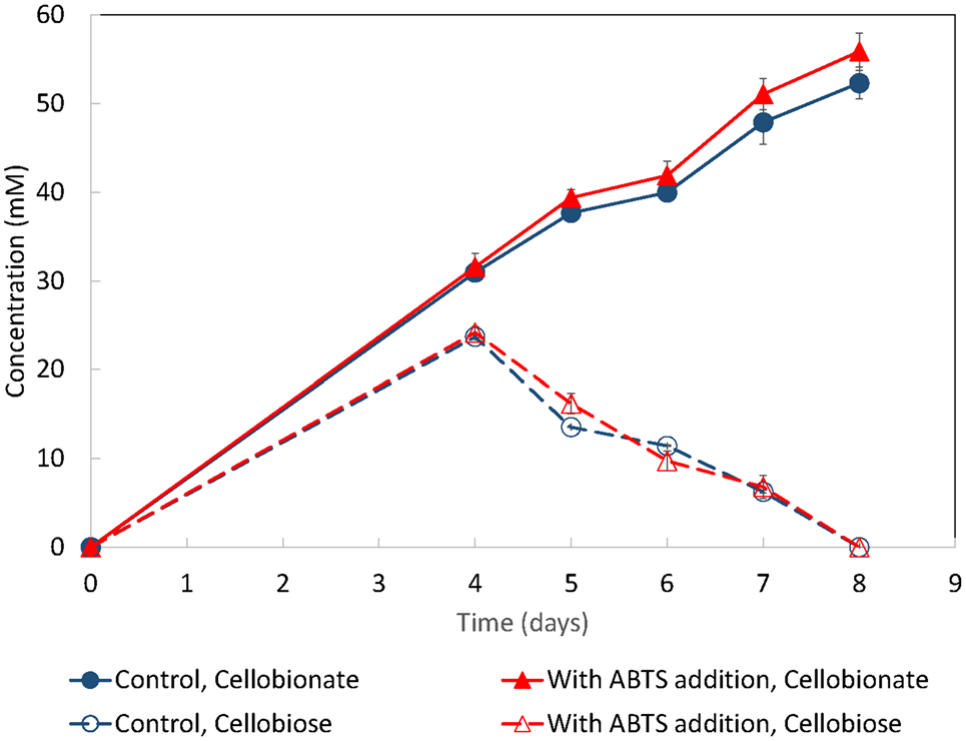
The effect of ABTS on cellobionate production from pretreated wheat straw using *N. crassa* HL10. The experiments were conducted in triplicate. Error bars indicate the standard deviations of sample replicates

The effect of ABTS on cellobionate production using NaOH-pretreated wheat straw as a substrate was investigated. As shown in Fig. 2, the cellobiose and cellobionate production profiles were very similar with or without ABTS addition, indicating that the effect of ABTS addition on CBA production was marginal when the NaOH-pretreated wheat straw was used as the substrate. Our prior study found that the natural lignin and its degradation products in wheat straw can serve as the redox mediator for the CDH-laccase system [31]. It seemed that the NaOH-pretreated lignin filled the same role. When lignin is present in the system, laccase can oxidize the lignin to oxidized lignin radicals and pass the electrons to oxygen as the final electron acceptor [32]. The lignin radical can receive the electrons from the reduced CDH, regenerating CDH and itself [31]. Adding ABTS led to only a marginal increase in cellobionate production, indicating that the system was no longer redox mediator limited. The cellobionate produced from 20 g/L cellulose equivalent wheat straw was about 52 mM, which is higher than that from 20 g/L Avicel.

### The effect of laccase on cellobionate production

To test the hypothesis that the CDH-redox mediator-laccase system in the HL10 fermentation system was laccase limited, we studied cellobionate production using strain HL10 with exogenous laccase and ABTS addition. 0.05 U/mL of laccase, or 0.05 U/mL of laccase plus 0.02 mM ABTS, were added to the wheat straw fermentation broth on day 4. As shown in Fig. 2, adding laccase alone led to faster cellobionate production. The strain *N. crassa* HL10 produced 58 mM cellobionate by day 6, while the control (the case without laccase addition) did not reach 57 mM cellobionate until day 8. Adding 0.02 mM of ABTS to the system besides laccase led to no further improvement in cellobionate production titer and rate.

These results indicated that laccase was the rate-limiting factor in cellobionate production using strain HL10 and pretreated wheat straw as the substrate. Additional laccase led to faster cellobionate production. However, the titer did not change. Further addition of ABTS besides laccase led to no noticeable improvement of CBA production, indicating that the redox mediator in the system was sufficient to support the bi-enzyme-catalyzed cellobionate production.

### The effect of cycloheximide addition on laccase production and cellobionate production by *N. crassa* HL10 on pretreated wheat straw

**Fig. 3:**
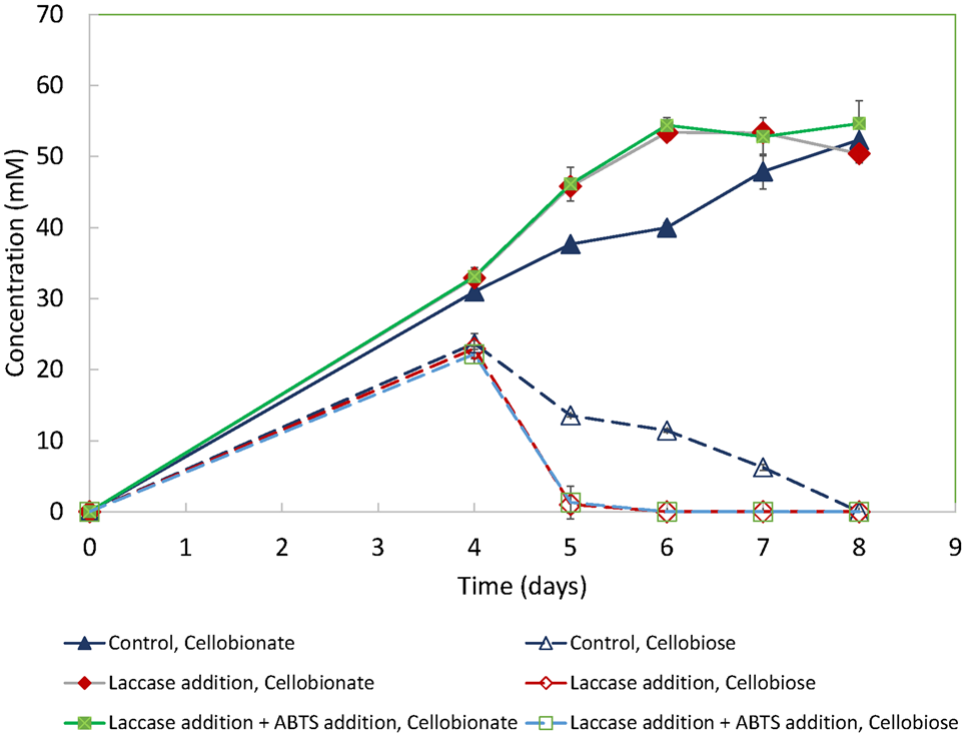
The effect of exogenous laccase addition on cellobionate production from pretreated wheat straw using *N. crassa* HL10. The experiments were conducted in triplicate. Error bars indicate the standard deviations of sample replicates

**Fig. 4:**
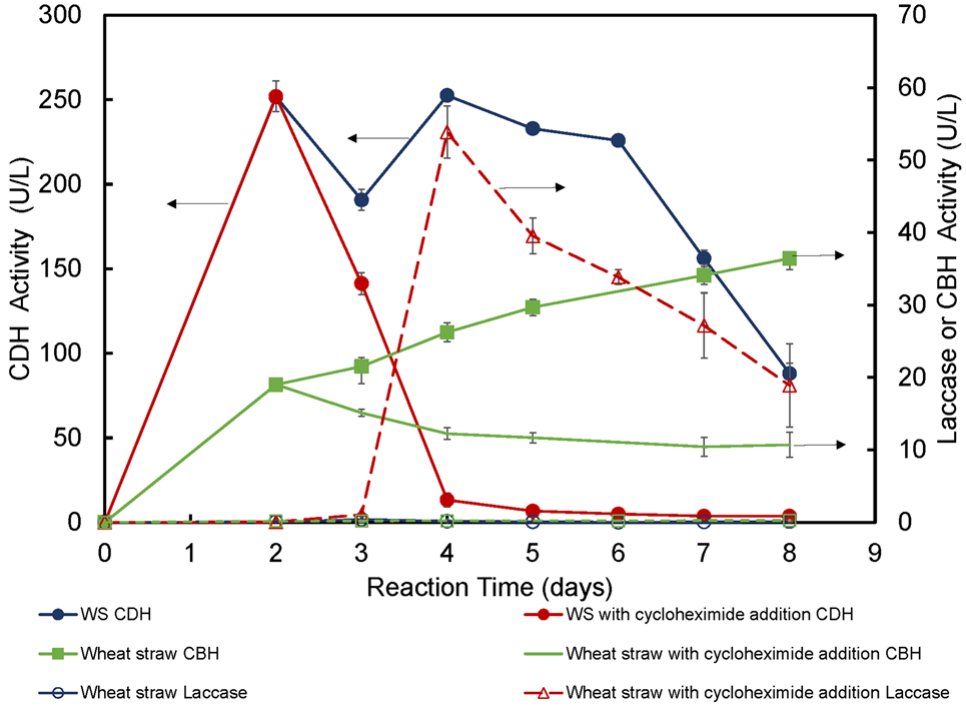
The effect of cycloheximide addition on CBH, CDH, and laccase production by *N. crassa* HL10 on pretreated wheat straw. The experiments were conducted in triplicate. Error bars indicate the standard deviations of sample replicates

**Fig. 5:**
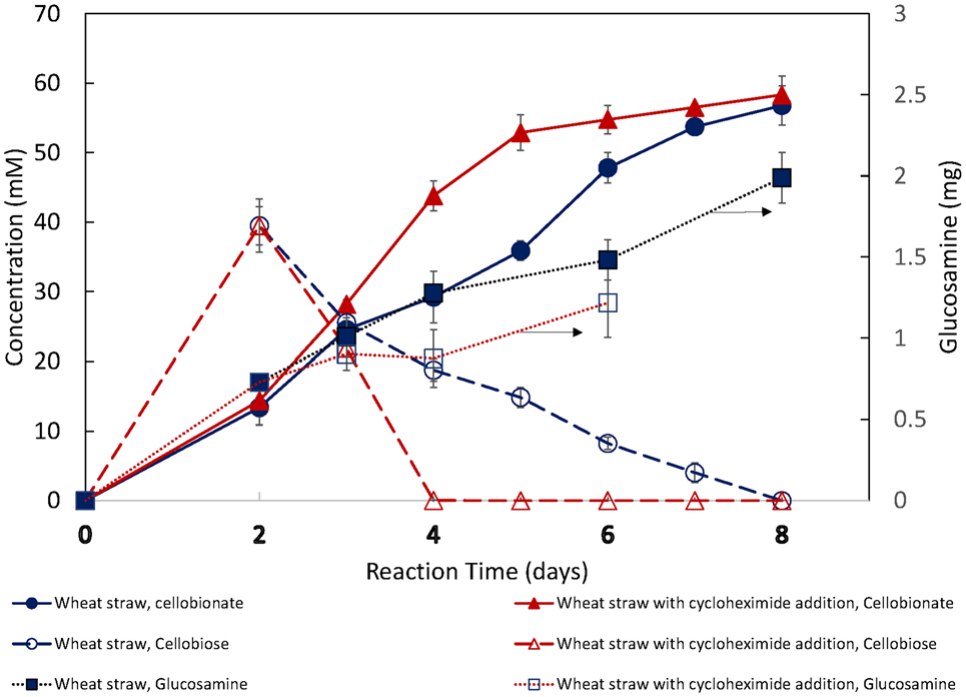
The effect of cycloheximide addition on fungal biomass and cellobionate production by *N. crassa* HL10 on pretreated wheat straw. The experiments were conducted in triplicate. Error bars indicate the standard deviations of sample replicates

The strain *N. crassa* HL10 was cloned with a heterologous laccase gene under a copper-inducible promoter. Fig. 3 It heterologously produced laccase at 30—80 U/L when the strain was grown on glucose with 0.8 mM copper sulfate added as the inducer [8]. When the same strain was grown on pretreated wheat straw, *N. crassa* HL10 (control) produced detectable laccase activity (< 0.38 U/L), as shown in Fig. 4. However, laccase activity was much lower than when the same strain was grown on glucose [8]. The laccase activity came from the expression of the heterologous laccase gene. *N. crassa* has a native laccase gene. However, it is not expressed when the strain grows vegetatively under the experimental conditions in this study [33, 34]. The native laccase gene is inducible when a protein synthesis inhibitor such as cycloheximide is present [33, 34]. Cycloheximide successfully induced laccase production in the *N. crassa* wild-type strain [33, 34]. The strain *N. crassa* F5*∆ace-1∆cre-1∆ndvB*, the parent strain of *N. crassa* HL10, produced up to 150 U/L of laccase upon the induction by 3 μM of cycloheximide when the strain was grown on glucose [24]. Cycloheximide also successfully induced native laccase production in strain *N. crassa* F5, a strain with multiple copies of β-glucosidase genes deleted, and improved the conversion of the lactose to lactobionic acid without exogenous laccase addition by this strain [35]. We applied a similar strategy to improve the cellobionate production by the strain *N. crassa* HL 10, which is a derivative of the strain *N. crassa* F5*∆ace-1∆cre-1∆ndvB,* using wheat straw as the substrate. Cycloheximide was added to the *N. crassa* HL10 fermentation on pretreated wheat straw at a final concentration of 3 μM on day 2. As shown in Fig. 4, apparent laccase activity was observed on day 3, one day after cycloheximide addition. The highest laccase activity of about 53.9 U/L was observed on day 4, and the laccase activity for the control and Avicel remained below 0.5 U/L. However, cycloheximide, a protein synthesis inhibitor, negatively affected CBH and CDH production. CBH activity accumulated to 36 U/L for the control. However, with cycloheximide addition, CBH began to decrease after 48 h and reached as low as 11 U/L at the end of the fermentation. (Fig. 5) CDH activity declined sharply to almost zero after cycloheximide addition, whereas CDH activity for the control (without cycloheximide addition) remained in the 87–252 U/L range. Cycloheximide also negatively affected fungal biomass production. However, due to higher laccase production in the fermentation broth, cellobiose conversion to cellobionate was accelerated despite the decline in CDH activity in the wheat straw system. The strain *N. crassa* HL10 produced about 57.0 mM of cellobionate on day 6 with cycloheximide addition, while the control (the case without cycloheximide addition) took 2 more days to reach that cellobionate concentration.

In a separate experiment conducted identically to the above experiment with cycloheximide addition on day 2 and the control, flasks were harvested after 6 days and 8 days, respectively, without intermediate sampling. The supernatant was collected for cellobionate and cellobiose measurements. The solid residues were analyzed for residual cellulose and xylan amount. The cellulose conversion for cycloheximide addition was about 97.3 ± 0.1% and the xylan conversion was about 98.0 ± 1.7% as shown in Table 1. The yield of the cellobionate from the consumed cellulose was about 97 ± 0.8%. About 92.0 ± 0.7% of the cellulose contained in the pretreated wheat straw was converted to cellobionate. The yield of the cellobionate from the consumed cellulose was close to the theoretical maximum. Cellulose conversion for the control was about 94.6 ± 0.3% and xylose conversion reached 99 ± 0.05%. There was no significant improvement in the cellobionate yield from consumed sugar between the control and cycloheximide addition.

**Table 1:** Percentages of cellulose hydrolyzed and converted to cellobionate for the HL10 strain grown on 20 g/L cellulose equivalent of NaOH-pretreated wheat straw with or without cycloheximide addition

Fermentation condition	Amount (g) of cellulose		Cellulose conversion (%)	Xylan conversion (%)	Yield (%) from consumed cellulose
	starting	residual			
Control	1.00	0.054 ± 0.003	94.6 ± 0.3	99 ± 0.1	96 ± 0.8
Cycloheximide addition	1.00	0.027 ± 0.001	97.3 ± 0.1	98 ± 1.7	97 ± 0.8

In a previous study, when *N. crassa* HL 10 was grown on 20 g/L Avicel as the substrate, the cellulose conversion was only 81% after 8-days of fermentation. The titer of cellobionate produced was about 47.4 mM. The cellobionate from the consumed cellulose was approximately 94.5%. About 76% of the cellulose contained in Avicel was converted to cellobionate [8]. When the strain *N. crassa* HL10 was grown on Avicel, the strain had a limited carbon source to support cell growth and enzyme production. Because the strain HL10 had multiple β-glucosidase genes and the cellobionate phosphorylase (*ndvB*) gene deleted from its genome, the strain can not use the main cellulose hydrolysis product, cellobiose, as the carbon source. Instead, it was fed on glucose generated by the side reaction of cellobiohydrolases on cellulose [36]. As a result, the fraction of cellulose that stayed un-converted was relatively high [8]. The cellobionate titer (57.0 mM) produced by *N. crassa* HL10 from the pretreated wheat straw, which contained 20 g/L cellulose equivalent, was significantly higher than that from Avicel.

It is worth noting that the presence of hemicellulose in wheat straw appeared to provide an additional carbon source that supported enhanced fungal growth and increased performance of the HL10 system compared to Avicel. *N. crassa* HL10 produced the full spectrum of hemicellulases to use the hemicellulose for cell growth and enzyme production [17]. Therefore, more cellulose was diverted to cellobionate production.

The NaOH-pretreated wheat straw is a more attractive substrate for cellobionate production than Avicel. Hemicellulose in the pretreated wheat straw provides an additional carbon source to support strain growth and enzyme production. Lignin serves as the redox mediator and removes the need for an exogenous artificial redox mediator. Under the optimized condition, cellulose and xylan conversions were very high (> 97%). The yield of cellobionate from the consumed cellulose was close to the theoretical maximum (97%). The heterologous laccase production in *N. crassa* HL10 was low when it was grown on pretreated wheat straw, which limited the cellobionate production rate. Cycloheximide addition successfully induced native laccase production and shortened the fermentation time. However, it harmed CBH, CDH, and fungal biomass production and also represented additional processing costs. It is desirable to find alternative strategies to improve laccase production. An alternative approach to increase laccase production is to de-repress native laccase production under vegetative growth conditions using genetic manipulation instead of protein inhibitors. The native laccase gene expression in *N. crassa* was regulated by a cross-pathway control gene *cpc-1* [37]. The *N. crassa* mutant lah-1 with increased expression of *cpc-1* produced extracellular laccase about three folders higher than when the wild type was induced with cycloheximide [37, 38]. We can potentially over-express the *cpc-1* gene in F5*∆ace-1∆cre-1∆ndvB* to construct a strain with the native laccase gene de-repressed. Another option is to improve the heterologous laccase production by engineering better promoter, secretion signal, and laccase gene.

## Conclusion

*N. crassa* HL10 produced cellobionate using Avicel as the substrate. However, high cellobionate yield was only achievable with exogenous redox mediators addition. Lignin in NaOH-pretreated wheat straw cellulosic biomass can effectively serve as a redox mediator, removing the need for an exogenous redox mediator. However, the low laccase activity produced by *N. crassa* HL10 remained a bottleneck for cellobionate conversion. Cycloheximide successfully induced a high level of laccase expression in *N. crassa* HL10. About 57 mM cellobionate was produced from the pretreated wheat straw containing 20 g/L cellulose in 6 days with 0.3 μM of cycloheximide on day 2. The cellulose conversion was about 97.3%. Cellobionate yield from the consumed cellulose was about 97%.

## Data Availability

The data supporting this study’s findings are available from the corresponding author ZF upon request.
